# TLR2 signaling regulates T cell exclusion in pancreatic ductal adenocarcinoma.

**DOI:** 10.1172/jci.insight.195329

**Published:** 2026-03-31

**Authors:** Jacqueline Plesset, Meredith L. Stone, John C. McVey, Heather Coho, Kelly Markowitz, Kayjana Coho, Jesse Lee, Anna S. Thickens, Devora Delman, Gregory L. Beatty

**Affiliations:** 1 Abramson Cancer Center, University of Pennsylvania, Philadelphia, Pennsylvania, USA.; 2 Division of Hematology-Oncology, Department of Medicine, and; 3 Department of Surgery, Perelman School of Medicine, University of Pennsylvania, Philadelphia, Pennsylvania, USA.

**Keywords:** Immunology, Inflammation, Oncology, Cancer, Cancer immunotherapy, T cells

## Abstract

Pancreatic ductal adenocarcinoma (PDAC) shows profound resistance to immunotherapy due to its immunosuppressive tumor microenvironment. Here, we studied the relationship between T cell infiltration and innate immune signaling in PDAC, identifying TLR2 as a key regulator of T cell exclusion. TLR2 expression correlated with T cell infiltration in both human and mouse PDAC tumors. Using genetic KO models and adoptive T cell transfer experiments, we found that TLR2 expression in both T cells and non–T cells contributes to T cell exclusion in PDAC. Notably, successful infiltration of adoptively transferred tumor-specific T cells required TLR2 deletion in both the transferred cells and the recipient host. The therapeutic implications of these findings are demonstrated through both genetic deletion and pharmacological inhibition of TLR2 using AAV-mediated and antibody-based approaches in murine models, resulting in decreased tumor growth and extended survival. Collectively, these findings identify TLR2 as a key modulator of T cell trafficking and immune suppression within the PDAC microenvironment, suggesting its potential as a therapeutic target for improving treatment outcomes.

## Abstract 2

This study identifies TLR2 as a driver of immune suppression in pancreatic cancer. Blocking TLR2 enables T cell entry, slows tumor growth, and may improve immunotherapy outcomes.

## Graphical abstract

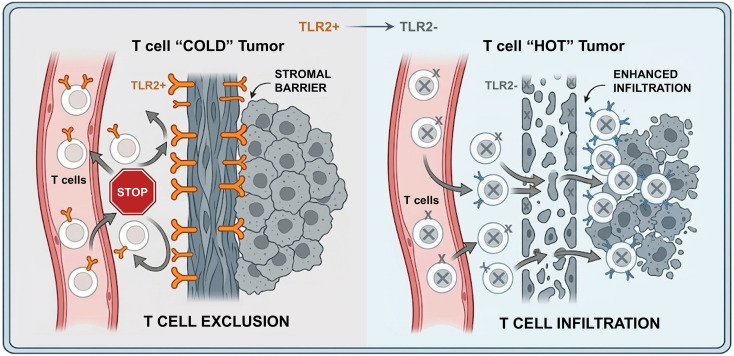

## Introduction

Pancreatic ductal adenocarcinoma (PDAC) remains one of the most challenging malignancies, with a dismal 5-year survival rate of 8% (1). Although survival rates have significantly improved for localized, surgically resected disease over the past 20 years, PDAC is often diagnosed at advanced stages, where therapeutic benefit with current standard of care chemotherapy is limited (2). Unlike in many other solid cancers including melanoma and non–small cell lung cancer, immunotherapy in PDAC has yet to provide reproducible clinical benefit to most patients. Notably, PDAC has shown remarkable resistance to cancer vaccines, immune checkpoint inhibitors, and adoptive T cell therapies (3–5). This resistance underscores the need to understand the unique tumor microenvironment (TME) and immune dynamics that coordinate immune escape in PDAC.

The success of immunotherapy is determined by a complex interplay between T cells, signaling pathways and the TME. Generally, patients with higher tumor T cell infiltration have improved responses to immunotherapy and overall survival. Additionally, infiltration of tumor-specific T cells correlates with better immunotherapy outcomes (6–8). However, the permissiveness of the TME to T cell infiltration is dependent on intratumoral antigen presenting cells, costimulatory factors that enhance T cell activation, cytokines that promote T cell expansion, and the degree of immunosuppression (6, 7, 9, 10). In PDAC, cancer cells coordinate a TME that actively inhibits these processes and undermines the potential of immunotherapy. This microenvironment is heavily dominated by immunosuppressive cells that secrete antiinflammatory cytokines, which suppress effector T cell activity and promote immune evasion (4, 5, 11). Furthermore, limited tumor antigenicity due to low tumor mutational burden restricts the neoantigen load necessary for effective immune recognition (12).

Improving outcomes with immunotherapy in PDAC requires a deeper understanding of the interconnected factors regulating T cell immunosurveillance. In this study, we investigate mechanisms underlying T cell exclusion in PDAC. Our findings show that PDAC infiltration by adoptively transferred tumor-specific T cells is enhanced in tumor models characterized by increased endogenous T cell infiltration. Through transcriptional profiling in both mice and humans, we identified TLR2 as increased in the TME and positively correlated with T cell infiltration. To determine the role of TLR2 in shaping TME biology, we examined its effect on T cell trafficking. Our findings show that TLR2 inhibits T cell infiltration into tumors. Additionally, we find that T cell–specific deletion of TLR2 in T cells is insufficient to overcome exclusion from the TME. Rather, T cell infiltration requires TLR2 deletion in both T and non–T cells. These findings are supported by pharmacological inhibition of TLR2, which suppresses tumor growth and improves overall survival in mice. These findings identify TLR2 as a key regulator of immune exclusion in PDAC and highlight its potential as a therapeutic to improve the immune permissiveness of the TME in PDAC.

## Results

### Preexisting T cell infiltration determines the efficacy of adoptive T cell therapy in PDAC.

**Figure 1:**
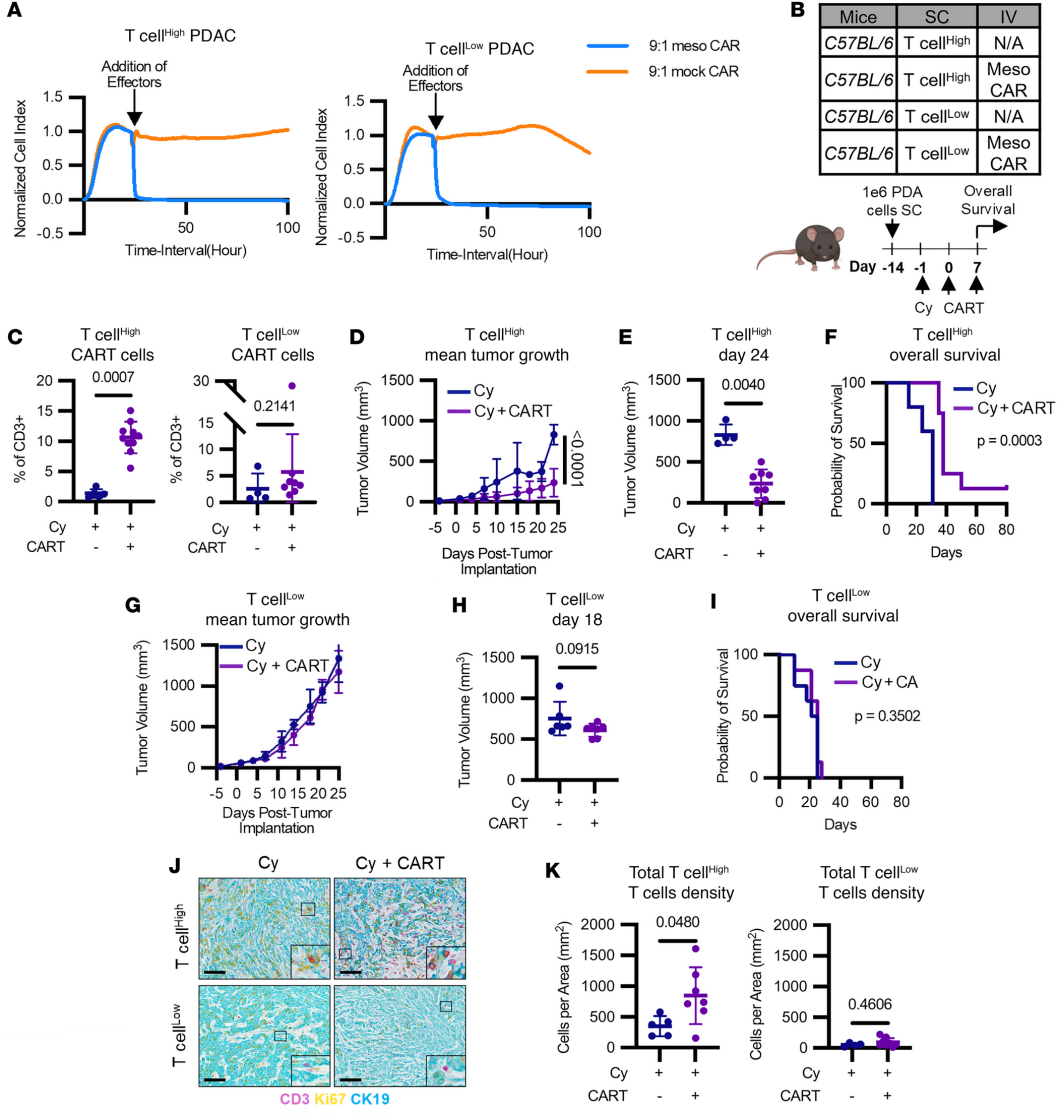
The immune microenvironment in PDAC determines adoptive T cell infiltration and efficacy. (**A**) In vitro xcelligence T cell^hi^ (2838c3) and T cell^lo^ (PDA.69) tumor cell survival after mesothelin or mock CAR T cells treatment (9:1 E:T). (**B**) Study design of **C**–**K**. T cell^hi^ or T cell^lo^ cells (1 × 10^6^) were implanted s.c. into WT mice (*n* = 5–8/group) on day –14. Mice received cyclophosphamide (120 mg/kg dose, i.p.) on day –1, followed by meso CAR-T cell infusions (5 × 10^6^ to 8 × 10^6^ cells/mouse, i.v.) on days 0 and 7. Data shown in **C**–**K** are representative of *n* = 2 experimental replicates. (**C**) Meso-CAR T cell trafficking in Cy versus Cy + CAR T cell–treated T cell^hi^ and T cell^lo^ tumors (Mann-Whitney *U* test performed). (**D**) Tumor growth curve (2-way ANOVA performed), (**E**) tumor volumes at day 24 (Mann-Whitney *U* test performed), and (**F**) overall survival curve (Mantel-Cox test performed) of mice bearing T cell^hi^ tumors treated with Cy or Cy + meso-CAR T cells. (**G**) Tumor growth curve (2-way ANOVA performed), (**H**) tumor volumes at day 18 (Mann-Whitney *U* test performed), and (**I**) overall survival curve (Mantel-Cox test performed) of mice bearing T cell^lo^ tumors treated with Cy or Cy + meso-CAR T cells. (**J**) Representative images of tumors stained for CD3 (pink), Ki67 (yellow) ,and CK19 (blue). Scale bar: 100 μm. (**K**) Analysis of T cell Density (CD3^+^) from J (Mann-Whitney *U* test performed).

To study the effect of T cell infiltration on the efficacy of immunotherapy, we investigated PDAC cell lines derived from the *LSL-Kras^G12D/+^ LSL-Trp53^R172H/+^ Pdx-1-Cre* (KPC) mouse model that coordinate a T cell inflamed (T cell^hi^) or noninflamed (T cell^lo^) TME. These cell lines have been characterized by flow cytometry and IHC, and have been used as models of T cell^hi^ and T cell^lo^ phenotypes in prior publications from our laboratory and others (13–15). Cell lines with both T cell^hi^ and T cell^lo^ phenotypes expressed comparable levels of the tumor-antigen mesothelin and were lysed in vitro by mesothelin-specific chimeric antigen receptor (meso-CAR) T cells (Figure 1A and Supplemental Figure 1, A and B; supplemental material available online with this article; https://doi.org/10.1172/jci.insight.195329DS1). We next tested the capacity of meso-CAR T cells to infiltrate and slow tumor growth in C57BL/6 (WT) mice implanted with T cell^hi^ and T cell^lo^ tumors. To enhance CAR T cell engraftment, tumor-bearing mice were treated on day –1 with a single 120 mg/kg dose of cyclophosphamide (Cy). This treatment induces transient lymphodepletion, reducing multiple lymphocyte populations and creating a more permissive immune environment for CAR T cell engraftement and expansion. On days 0 and 7, mice were treated with or without meso-CAR T cells (Figure 1B). Meso-CAR T cells were found to infiltrate T cell^hi^ but not T cell^lo^ tumors (Figure 1C). Consistent with this, meso-CAR T cell treatment slowed tumor growth and prolonged overall survival in T cell^hi^ tumors (Figure 1, D–F, and Supplemental Figure 1, C and D). However, in T cell^lo^ tumors, meso-CAR T cell therapy did not significantly affect tumor growth or have a significant effect on overall survival (Figure 1, G–I, and Supplemental Figure 1, E and F). Further analysis of the tumors showed that mice bearing T cell^hi^ tumors had an increase in T cell density after Meso-CAR T cell therapy that was not observed in mice bearing T cell^lo^ tumors (Figure 1, J and K). Analysis of meso-CAR T cells in the blood showed comparable CAR T cell frequencies in both tumor models, indicating that the observed therapeutic differences were not attributable to differences in T cell engraftment or persistence in vivo (Supplemental Figure 1, G and H). Together, these findings support a role for the preexisting TME, rather than insufficient tumor-specific T cells, as a rate limiting determinant of the efficacy of immunotherapy in PDAC.

### TLR2 expression correlates with T cell infiltration in human and mouse PDAC.

**Figure 2:**
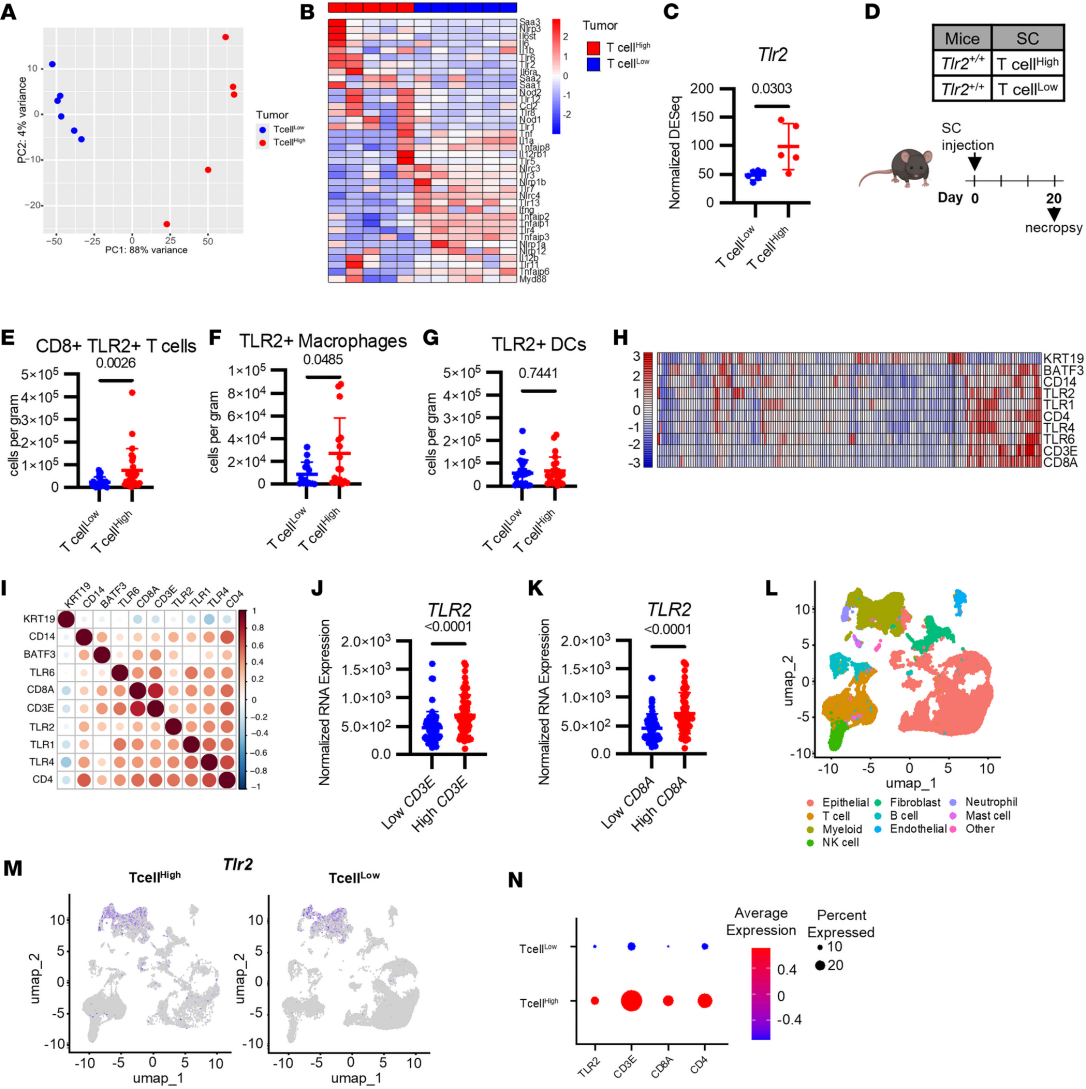
Innate immune signaling molecules correlate with increased tumor immune infiltration in PDAC. (**A**) PCA plot of T cell^hi^ versus T cell^lo^ bulk tumor RNA-seq. (**B**) Innate signaling pathway heatmap of T cell^hi^ versus T cell^lo^ bulk tumor RNA-seq. (**C**) Normalized TLR2 DESeq expression from bulk RNA-seq data (Mann-Whitney *U* test performed). (**D**) Study design of T cell^hi^ versus T cell^lo^ end point study. T cell^hi^ (2838c3) or T cell^lo^ (PDA.69) cells (1 × 10^6^) were implanted s.c. into *Tlr2^+/+^* mice (*n* = 8/group) on Day 0. Tumors were extracted with necropsy on day 20. (**E**) CD8^+^TLR2^+^ T cell (CD45^+^CD3^+^CD8^+^TLR2^+^) per gram. Data shown is *n* = 3 experimental replicates (Mann-Whitney *U* test performed). (**F**) TLR2^+^ macrophages (CD45^+^CD11b^+^F480^+^CD3^–^CD19^–^Ly6G^–^ TLR2^+^) per gram. Data shown is *n* = 2 experimental replicates (Mann-Whitney *U* test performed). (**G**) TLR2^+^ DCs (CD45^+^CD11c^+^F480^–^CD3^–^CD19^–^Ly6G^–^ TLR2^+^) per gram. Data shown is *n* = 3 experimental replicates (Mann-Whitney *U* test performed). (**H**) Heatmap of TCGA data of *n* = 172 patients with pancreas cancer. (**I**) Gene correlation plot of TCGA data. (**J** and **K**) Normalized *TLR2* RNA Expression of high (quartiles 3 and 4) versus low (quartiles 1 and 2) *CD3E* and *CD8A* gene expression from TCGA data. (**L**) Labeled UMAP of single cell RNA-seq data taken from 6 PDAC patients. (**M**) UMAP of *TLR2* expression in single cell RNA-seq data divided into T cell^hi^ versus T cell^lo^ patients. T cell^hi^ patients: ≥30% of cells found in their tumors are T cells. T cell^lo^ patients: ≤10.5% of cells in their tumors are T cells (22). (**N**) Dot plot of *TLR2*, *CD3E*, *CD8A*, and *CD4* genes from single cell RNA-seq dataset.

To identify factors within the TME that contribute to T cell infiltration versus exclusion, we analyzed RNA-seq obtained from tumors orthotopically implanted with T cell^hi^ and T cell^lo^ cell lines (Figure 2A). Differential expression analysis revealed 3,203 genes upregulated in T cell^hi^ tumors and 2,959 upregulated genes in T cell^lo^ tumors (Supplemental Figure 2A and Supplemental Table 4). We studied innate signaling pathways (16), including TLR pathways, and their relationship with CD3^+^ T cell infiltration (Figure 2B). Among a set of 38 genes related to these pathways (Figure 2B), we identified 8 differentially expressed genes. Notably, among the TLR pathways, only *TLR1* and *TLR2* were significantly different with increased expression detected in T cell^hi^ tumors compared with T cell^lo^ tumors (Figure 2C and Supplemental Figure 2, B–K). Given that *TLR1* is known to form a heterodimer with *TLR2* (17, 18), we focused our subsequent studies on TLR2. We examined tumor-infiltrating immune cells for TLR2 expression by flow cytometry. This analysis revealed a significant increase in TLR2^+^ CD8^+^ T cells and TLR2^+^ F4/80^+^ macrophages in T cell^hi^ tumors compared with T cell^lo^ tumors. No difference was observed in TLR2^+^CD11c^+^F4/80^–^ DCs (Figure 2, D–G). To investigate potential TLR2 ligands within tumors, we assessed expression of multiple known TLR2 ligands and found *Hmgb1* and *Has3* significantly elevated in T cell^lo^ compared with T cell^hi^ tumors (Supplemental Figure 2L). Together, these findings show that TLR2 expression is increased in T cell^hi^ versus T cell^lo^ tumors and is present on multiple immune cell subsets within the TME.

We next analyzed PDAC tumor samples from The Cancer Genome Atlas (TCGA). Similar to our findings in mice, *TLR2* expression positively correlated with T cell markers (*CD3E*, *CD8A*, and *CD4*) in tumors from patients with surgically resected or biopsied pancreas cancer (Figure 2, H and I, and Supplemental Table 5). *TLR2* expression also correlated with myeloid cell–associated genes (*CD14*, *BATF3*), supporting its presence on various immune cells within the TME (18–20). Consistent with findings in mice, *TLR1* also correlated with T cell markers in human PDAC. Additionally, *TLR6* significantly correlated with T cell markers in human PDAC (Supplemental Figure 3, A–I), though this was not significant in mice (Supplemental Figure 2). Notably, both TLR1 and TLR6 form heterodimers with TLR2. Tumors with high T cell levels (*CD3E* and *CD8A*) also exhibited significantly higher *TLR2* expression compared with those with low T cell levels (Figure 2, J and K). Furthermore, among patients with high T cell infiltration, those with concomitant high TLR2 expression showed a trend toward increased median survival (691 days versus 518 days), though this difference was not statistically significant (*P* = 0.07; Supplemental Figure 3J).

To further test the relationship between *TLR2* and T cell infiltration, we categorized human pancreatic cancer samples with publicly available single-cell RNA-seq data (13) into T cell^hi^ (>30% T cells) or T cell^lo^ (<10.5% T cells). UMAP clustering revealed enriched *TLR2* expression on myeloid cells in the T cell^hi^ group compared with the T cell^lo^ group (Figure 2, L and M). Dot plots confirmed increased TLR2 expression specifically in T cell^hi^ samples (Figure 2N). We next analyzed TLR2 signaling in T cells from T cell^hi^ versus T cell^lo^ samples. We calculated a TLR1/2 signaling score at single-cell resolution using the AddModuleSocre function in Seurat, applying 115 genes from the “Reactome Toll Like Receptor TLR1 TLR2 Cascade” gene set as a measure of relative TLR1/2 pathway activity. T cells from T cell^lo^ tumors showed significantly higher TLR1/2 signaling scores, including across subsets (CD8, CD4, effector CD8, and Tregs), compared with T cell^hi^ tumors (Supplemental Figure 3, K–M). Taken together, these data identify a strong correlation between *TLR2* expression and T cell infiltration in PDAC across both human and mouse tumors and suggest that increased TLR2 signaling in T cells may restrict their infiltration into PDAC tumors.

### TLR2 regulates T cell activation and tumor infiltration.

**Figure 3:**
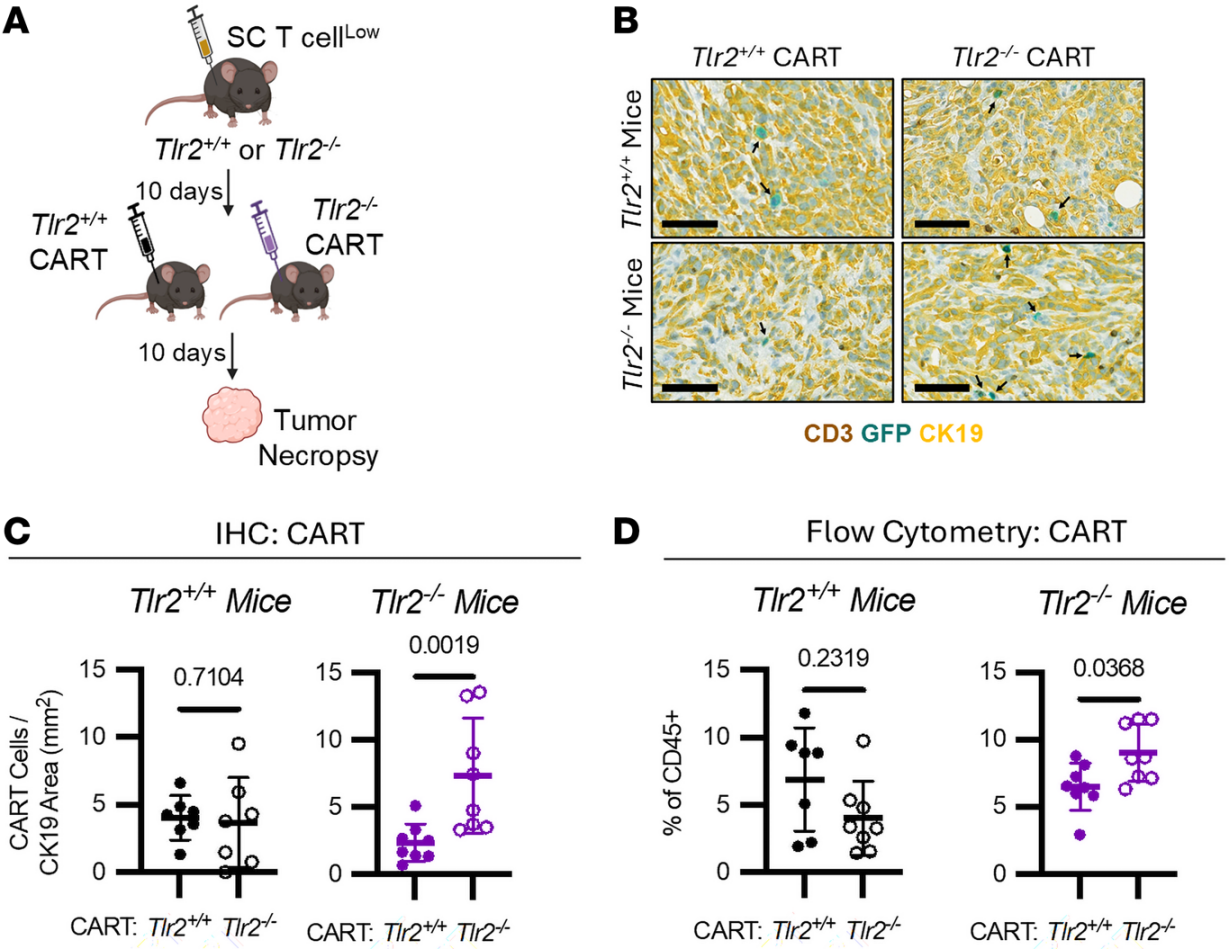
TLR2 expression in both T cells and host cells regulates T cell infiltration in PDAC. (**A**) Study design of **B**–**D**. T cell^lo^ (PDA.69) cells where s.c. implanted into *Tlr2^+/+^* and *Tlr2^–/–^* mice on day –10. On Day –1, mice received Cy (120 mg/kg dose, i.p.) and then *Tlr2^+/+^* or *Tlr2^–/–^* CART cells on day 0 with necropsy 10 days later. (**B**) Representative images of tumors stained for GFP (teal), CD3 (brown), and CK19 (yellow). Scale bar: 60 μm. (**C**) Analysis of CART cell densities (GFP stained) from B (*n* = 7–8/group, Mann-Whitney *U* test performed). (**D**) Analysis of CART cell percentages in the tumors via flow cytometry (*n* = 7–8/group, Mann-Whitney *U* test performed). In **C** and **D**, black represents *Tlr2^+/+^* mice while purple represent *Tlr2^–/–^* mice. Open circles represent *Tlr2^–/–^* CAR T cells, while closed circles represent *Tlr2^+/+^* CAR T cells.

We previously showed that serum amyloid A proteins 1 and 2 (SAA), which are acute phase reactants secreted by hepatocytes in the liver in the setting of PDAC, engage TLR2 to restrict endogenous T cell infiltration into PDAC tumors (21). Specifically, endogenous T cell infiltration into PDAC tumors was increased in *Tlr2^–/–^* compared with *Tlr2^+/+^* mice (21). Based on this, we assessed whether TLR2 expression affects meso-CAR T cell trafficking into PDAC tumors. To do this, we studied *Tlr2^+/+^* and *Tlr2^–/–^* mice implanted with T cell^lo^ tumors. Tumor-bearing mice were then treated with Cy on day –1, followed by i.v. injection of *Tlr2^+/+^* or *Tlr2^–/–^* meso-CAR T cells on day 0 (Figure 3A and Supplemental Figure 4A). IHC and flow cytometry analyses revealed no effect of TLR2 expression on meso-CAR T cell trafficking in *Tlr2^+/+^* mice (Figure 3, B–D), indicating that deleting TLR2 in CAR T cells alone was not sufficient to overcome T cell exclusion. However, in *Tlr2^–/–^* mice, deletion of TLR2 in meso-CAR T cells significantly enhanced their infiltration into tumors compared with *Tlr2^+/+^* meso-CAR T cells (Figure 3, B–D). Additionally, *Tlr2^–/–^* mice had more *Tlr2^–/–^* meso-CAR T cell infiltration compared with *Tlr2^+/+^* mice (Supplemental Figure 4, B and C). These findings indicate that TLR2 expression on both CAR T cells and host cells cooperatively regulate T cell infiltration into tumors, with deletion of TLR2 in both compartments required for optimal T cell infiltration.

We next hypothesized that SAA might engage TLR2 on CAR T cells to inhibit their trafficking into PDAC tumors. To test this, we assessed meso-CAR T cell infiltration into T cell^lo^ tumors implanted into *Saa^+/+^* and *Saa^–/–^* mice (Supplemental Figure 4, D–H). While endogenous T cells were significantly increased in *Saa^–/–^* mice, consistent with our prior data (21), no difference in CAR T cell infiltration into PDAC tumors was observed. This finding suggests a role for additional TLR2 ligands, beyond SAA, in specifically regulating meso-CAR T cell trafficking.

### TLR2 is a determinant of response to therapy in PDAC.

**Figure 4:**
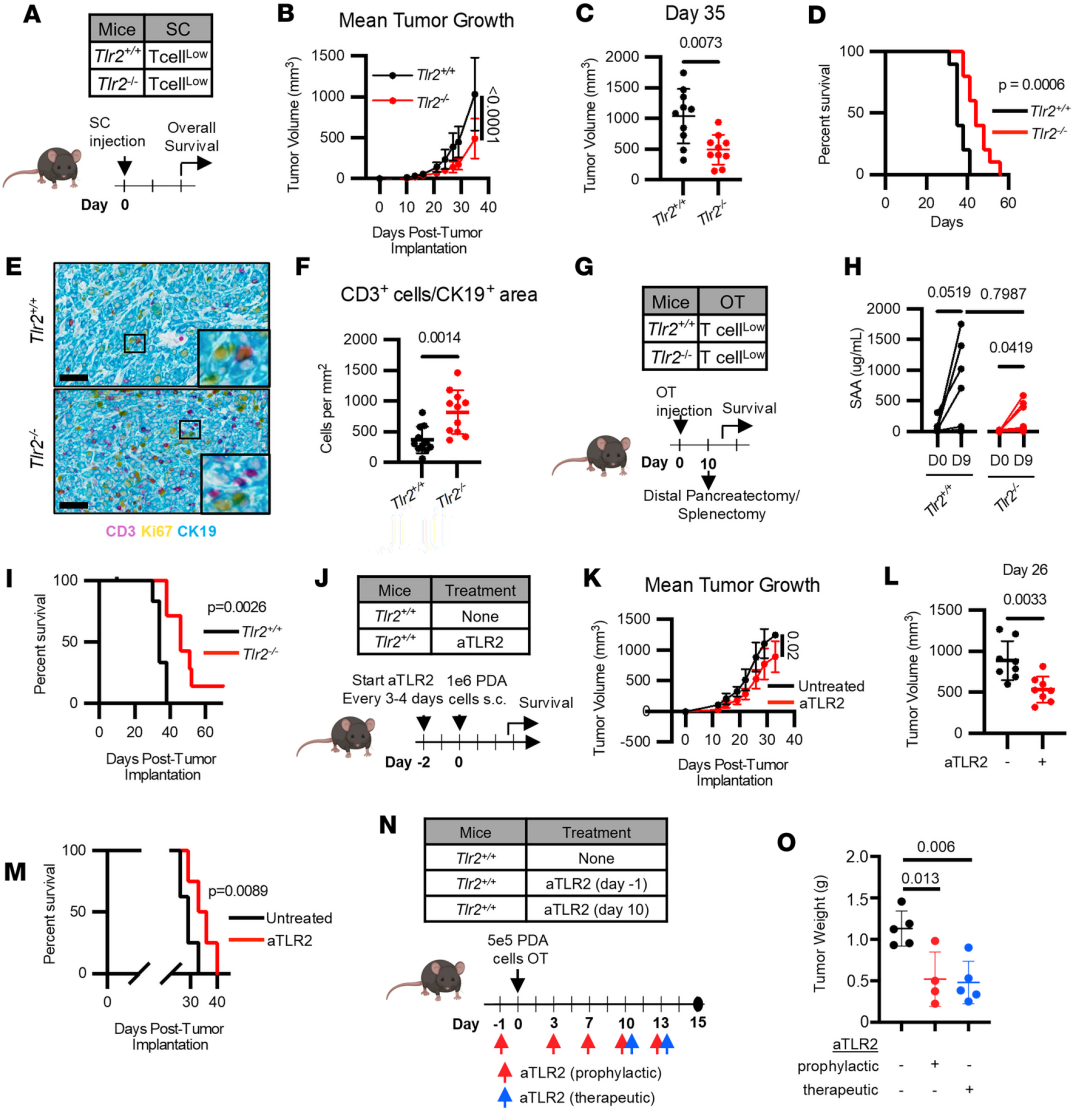
TLR2 is a therapeutic target in PDAC. (**A**) Study schematic for **B**–**F**. T cell^lo^ (PDA.69) cells (1 × 10^6^) were implanted s.c. into *Tlr2^+/+^* or *Tlr2^–/–^* mice (*n* = 10/group) on day 0. Data are representative of 1 experiment. (**B**) Mean tumor growth (2-way ANOVA test performed). (**C**) Tumor size day 35 (Mann-Whitney *U* test performed). (**D**) Overall survival (Mantel-Cox test performed). (**E**) Representative images of tumors stained for CD3 (pink), Ki67 (yellow), and CK19 (blue). Scale bar: 50 μm. (**F**) Analysis of T cell (CD3 stained) per tumor area (CK19 stained) from **E** (Mann-Whitney *U* test performed). (**G**) Study schematic for **H** and **I**. T cell^lo^ (PDA.69) cells (1 × 10^5^) were orthotopically implanted into *Tlr2^+/+^* (*n* = 8) or *Tlr2^–/–^* (*n* = 7) mice. Ten days later, tumors were surgically resected. Mice were monitored for overall survival. (**H**) SAA serum level measured by ELISA (Wilcoxon matched pairs signed rank test performed, or Mann-Whitney *U* test performed for unpaired groups). Data shown are from *n* = 2 experimental replicates. (**I**) Overall survival (Mantel-Cox test performed). Data are representative of *n* = 3 experimental replicates. (**J**) Study schematic for **K**–**M**. *Tlr2^+/+^* mice were treated with aTLR2 (0.2 mg/dose, Sino) on day –2. On day 0, mice were s.c. challenged with T cell^lo^ (PDA.69) cells (1 × 10^6^). (**K**) Mean tumor growth curves (2-way ANOVA test performed). (**L**) Tumor volume day 26 (Mann-Whitney *U* test performed). (**M**) Overall survival (Mantel-Cox test performed). (**N**) Study schematic for **O**. On day 0, *Tlr2^+/+^* mice were orthotopically implanted with 5 × 10^5^ T cell^lo^ (6694c2) cells. aTLR2 (0.2 mg/dose) was administered i.p. every 3–4 days starting on day –1 (prophylactic, red arrows) or day 10 (therapeutic, blue arrows). (**O**) Tumor weight on day 15 after tumor implantation (1-way ANOVA with Tukey correction).

Our findings suggest a role for TLR2 in regulating T cell exclusion in PDAC, thereby prompting us to investigate the therapeutic effect of targeting TLR2. To assess the effect of TLR2 signaling on survival, we implanted T cell^lo^ tumor cells into *Tlr2^+/+^* and *Tlr2^–/–^* mice (Figure 4A). *Tlr2^–/–^* mice exhibited significantly slower tumor growth and increased survival compared with *Tlr2^+/+^* mice (Figure 4, B–D, and Supplemental Figure 5A). This improved outcome was associated with a significant increase in tumor-infiltrating T cells in *Tlr2^–/–^* mice (Figure 4, E and F). T cell profiling by IHC showed similar levels of intratumoral CD4^+^ cells but increased CD8^+^ cells in *Tlr2^–/–^* mice compared with *Tlr2*^+/+^ mice (Supplemental Figure 5, B–D). Additional myeloid cell profiling revealed a reduction in CD11b^+^ myeloid cells in *Tlr2^–/–^* mice (Supplemental Figure 5, E and F). Among CD11b^+^ cells, there were comparable levels of macrophages and DCs but a reduction in Ly6c^+^ monocytes (Supplemental Figure 5, G–I). We next tested the role of TLR2 in surgical outcomes by performing distal pancreatectomy/splenectomy on *Tlr2^+/+^* versus *Tlr2^–/–^* mice with orthotopically implanted T cell^lo^ tumors (Figure 4G). Our prior studies showed a role for SAA in reduced survival after surgery but did not define the role of TLR2 (21). Here, SAA protein levels increased in both *Tlr2^+/+^* and *Tlr2^–/–^* mice following tumor implantation, indicating that TLR2 is not necessary for SAA secretion in vivo (Figure 4H). Despite this, *Tlr2^–/–^* mice still had significantly increased survival (Figure 4I). This finding is consistent with SAA-TLR2 signaling in nonmalignant cells driving tumor progression.

Based on the improved survival observed in mice lacking TLR2, we next tested the therapeutic potential of pharmacologically targeting TLR2. We engineered a liver-targeted adeno-associated viral vector (AAV.2/8) expressing an HA-tagged anti-TLR2 scFv compared with GFP under the control of the hepatocyte-specific TBG promoter (Supplemental Figure 6A). Following i.v. administration, successful vector delivery and expression was confirmed 7 days later by detecting the HA tag in the liver, serum, and spleen of *Tlr2^+/+^* mice that had received aTLR2.AAV (Supplemental Figure 6, B–E). Consistent with functional TLR2 inhibition, splenocytes isolated from aTLR2.AAV-treated mice stimulated ex vivo with the TLR2 ligand PAM3CSK4 displayed reduced IL-6 secretion compared with splenocytes from untreated mice (Supplemental Figure 6, B and F). We next administered GFP.AAV or aTLR2.AAV to *Tlr2^+/+^* mice prior to implantation of T cell^lo^ tumor cells (Supplemental Figure 6G). Similar to our observations in *Tlr2^–/–^* mice, tumors grew significantly slower and survival was increased in aTLR2.AAV-treated mice compared with GFP.AAV-treated controls (Supplemental Figure 6, H–J). Finally, we engineered a full-length aTLR2 antibody on an IgG1 backbone. *Tlr2^+/+^* mice were treated with the aTLR2 antibody starting 2 days prior to T cell^lo^ tumor cell implantation, and treatment continued every 3–4 days for 21 days (Figure 4J). Mice receiving aTLR2 showed significantly slower tumor growth and extended survival compared with untreated mice (Figure 4, K–M). To assess therapeutic efficacy, mice were orthotopically implanted with a T cell^lo^ tumor and treated with aTLR2 starting 1 day prior to or 10 days after implantation. Prophylactic and therapeutic aTLR2 similarly reduced tumor growth compared with untreated controls (Figure 4, N and O). Collectively, these data identify TLR2 as a therapeutic target for PDAC. In summary, our findings suggest TLR2 as an immune checkpoint that regulates T cell infiltration and tumor growth in PDAC. Our studies support strategies that intervene on TLR2 as an approach to reverse T cell exclusion in PDAC and potentially enhance the efficacy of immunotherapies.

## Discussion

In this study, we identify a key role for TLR2 in regulating T cell infiltration into PDAC tumors. We find that preexisting T cell infiltration determines the success of adoptive T cell therapy and that TLR2 expression correlates with T cell infiltration in both human and mouse PDAC. Our findings suggest that TLR2 expression on both T cells and non–T cells determines T cell infiltration into PDAC. Furthermore, we show that targeting TLR2 can improve survival outcomes in PDAC models. Taken together, our data identify TLR2 as an immune checkpoint and potential therapeutic target in PDAC.

Our findings show that the immune phenotype of PDAC tumors — specifically, whether they are T cell–inflamed or noninflamed — is a critical determinant of the efficacy of adoptive T cell therapy efficacy (Figure 1, D–I). Consistent with this, mice bearing T cell^hi^ tumors exhibited greater T cell infiltration after CAR T cell therapy, an effect not observed in T cell^lo^ tumors (Figure 1, J and K), suggesting that tumor immunogenicity critically influences CAR T cell efficacy. This observation has important implications for patient enrichment strategies in clinical trial designs, potentially allowing for the identification of patients most likely to benefit from CAR T cell therapy and other immunotherapies. Notably, T cell–inflamed tumors in mouse models have also shown sensitivity to immune checkpoint blockade targeting CTLA4 and PD1 (13, 22). Moreover, PDAC tumors defined by increased T cell infiltration and immunologically active cellular communities associate with favorable outcomes in PDAC (15, 21, 23). Together, these data suggest that the immune phenotype of PDAC could serve as a biomarker for response to various immunotherapeutic approaches.

Our results demonstrate that TLR2 expression is increased in T cell^hi^ tumors compared with T cell^lo^ tumors, with significant correlations between TLR2 expression and T cell markers in both mouse models and human PDAC samples. Interestingly, we observed an increased presence of TLR2-expressing T cells in T cell–inflamed tumors in both mice and humans. This seemingly paradoxical finding likely reflects the fact that, in the absence of TLR2 ligands, more TLR2-expressing T cells are able to infiltrate tumors. In support of this, we observed increased TLR1/2 signaling in T cells of T cell^lo^ human PDAC samples compared with T cell^hi^. This relationship suggests that TLR2 plays a pivotal role in shaping the tumor immune microenvironment, with its effect dependent on the presence or absence of its ligands. Consistent with this, we detected higher expression of known endogenous TLR2 ligands, *Hmgb1* and *Has3*, in T cell^lo^ tumors compared with T cell^hi^ tumors. *Hmgb1* expression is of particular interest due to its known ability to engage TLR2 on immune cells and impair T cell infiltration and the efficacy of immune checkpoint therapy in breast and non–small cell lung cancer tumor models (24). We also observed TLR2 expression on multiple immune cell subsets within the TME, including CD8^+^ T cells and F4/80^+^ macrophages, indicating a complex interplay between TLR2 signaling and various immune cell populations. These findings underscore the nuanced role of TLR2 in modulating the immune landscape of PDAC tumors, and they highlight the importance of considering both receptor expression and ligand availability when interpreting TLR2’s effects on T cell infiltration.

The mechanisms of TLR2-mediated immune regulation in PDAC are complex and multifaceted. In our studies, we investigated SAA because of our previous work identifying it as a TLR2 ligand that restricts T cell infiltration into PDAC tumors (21). Our prior findings suggest that this T cell exclusion is due to TLR2 inhibition of dendritic cell maturation and subsequent T cell priming, which typically occurs in lymph nodes (21). However, our current studies reveal interesting differences between SAA-KO mice and TLR2-KO mice. While both endogenous T cells and TLR2-KO CAR T cells infiltrate tumors in mice lacking TLR2, only endogenous T cells infiltrate in SAA-KO mice. This discrepancy suggests differential requirements for CAR T cells versus conventional endogenous T cells. In this regard, CAR T cells do not ordinarily recognize their target on DCs and are less dependent on these cells for infiltration into tumors, although DCs may be important for sustaining CAR T cell persistence within tumors (25). Thus, our findings highlight a fundamental difference between CAR T cells and endogenous TCR T cells in cancer immunology. Importantly, our data also indicate that merely eliminating TLR2 on CAR T cells is insufficient to promote their infiltration, suggesting that TLR2 plays a broader role in modulating the immune permissiveness of the TME.

The improved survival observed in *Tlr2^–/–^* mice and the therapeutic efficacy of TLR2 inhibition using both AAV-mediated and antibody-based approaches underscore the potential of targeting TLR2 in the treatment of PDAC. These findings are particularly significant given the historically poor response of PDAC to immunotherapies. By identifying TLR2 as an immune checkpoint that regulates T cell infiltration and tumor growth, our study provides a potential target for enhancing the immunotherapy efficacy. Importantly, aTLR2 therapy demonstrated the ability to impair established tumor growth, suggesting clinical utility even after tumor development. In the neoadjuvant setting prior to surgical resection, TLR2 inhibition — as demonstrated by genetic knockout models — may be particularly effective by modulating the TME to enhance immunogenicity through increased T cell infiltration, thereby limiting postoperative tumor recurrence. This contrasts with the therapeutic setting, where TLR2 inhibition may primarily impair tumor growth through distinct mechanisms beyond T cell infiltration, potentially through modulating myeloid cell recruitment into tumors (Supplemental Figure 7, A–C).

While our study provides compelling evidence for the role of TLR2 in PDAC immunotherapy resistance, several questions remain. Future research should focus on identifying the specific TLR2 ligands involved in regulating CAR T cell infiltration, investigating the differential effects of TLR2 signaling on various immune cell subsets within the TME, and determining potential synergies between TLR2 inhibition and other immunotherapeutic approaches.

In conclusion, our study identifies TLR2 as a key regulator of immune exclusion in PDAC and highlights its potential as a therapeutic target to improve the immune permissiveness of the TME. By enhancing T cell infiltration and tumor control, TLR2 inhibition may offer a promising strategy to overcome the limitations of current immunotherapies in PDAC. These findings provide a strong rationale for further investigation of TLR2-targeted approaches in combination with existing immunotherapies for the treatment of PDAC.

## Methods

### Sex as a biological variable.

Studies included both male and female mice, with all groups sex and age matched. Similar findings were observed in both sexes, indicating no sex-specific effects.

### Cell lines.

PDA.69 and 2838c3 were used in s.c. tumor models. PDA.69 is derived from primary PDAC tumors that spontaneously develop in KPC mice as previously described (21). 2838c3 is derived from KPCY tumors as previously described (13). HEK Blue mTLR2 SEAP reporter cell line was purchased from InvivoGen and used according to manufacturer’s instructions. All tumor cell lines were cultured in DMEM (Corning) with 10% FBS (VWR), 83 μg/mL gentamicin (Thermo Fisher Scientific), and 1% GlutaMAX (Thermo Fisher Scientific) at 37°C and 5% CO_2_. Cell lines passaged fewer than 20 times with greater than 85% viability (confirmed with trypan blue staining) were used in experiments. All cell lines used tested negative for *Mycoplasma* contamination at the Cell Center Services Facility at the University of Pennsylvania.

### Animal studies.

C57BL/6J and *Tlr2^–/–^* (TLR2^tm1Kir^) mice were obtained from The Jackson Laboratory. All transgenic mice were bred and maintained within the animal facility of the University of Pennsylvania. *Saa1* and *Saa2* double knockout (*Saa^–/–^*) mice were gifted from Maria C. de Beer, Frederick C. de Beer, and Nancy R. Webb (University of Kentucky, Lexington, Kentucky) (21, 26). In general, mice were monitored 3 times per week for general health and euthanized early based on defined endpoint criteria including tumor volume ≥ 1,000 mm^3^, ascites, lethargy, loss of ≥ 10% body weight, or other signs of sickness or distress.

Mice were randomized in an unblinded fashion. Sample sizes were estimated based on pilot experiments and chosen to ensure an adequate number of mice per group for statistical analysis.

In s.c. tumor studies, tumor cells in 150 mL sterile PBS were injected with a 30 gauge needle in the left flank. Tumors were calipered twice a week, and mice were euthanized when tumor volume reached ≥ 1,000 mm^3^.

Orthotopic tumor cell injections were performed under anesthesia. Mice underwent a laparotomy (5–10 mm incision) in the upper left quadrant of the abdomen. The pancreas was exteriorized, and pancreatic tumor cells (5 × 10^5^ cells in 50 mL sterile PBS) were injected into the tail of the pancreas. Successful injection was confirmed by the formation of a liquid bleb. The pancreas was then gently returned to the peritoneal cavity. For studies involving pancreatectomy/splenectomy, after 10 days of tumor growth, mice were anesthetized with continuous isoflurane, analgesia was administered, and the depth of anesthesia was carefully evaluated. The abdomen was sterilized, and a second laparotomy was performed in the upper left quadrant. The distal pancreas and spleen were resected using electrocautery and hemostatic clips, maintaining meticulous hemostatic control throughout the procedure. All surgical closures were performed using absorbable sutures (Midwest Veterinary) for the peritoneum and wound clips (Braintree Scientific) for the skin.

For injection of AAV, mice were i.v. injected with 100 μL of a viral preparation containing 1 × 10^12^ viral genomes (vg) encoding murine TLR2 (aTLR2.AAV) or green fluorescent protein (GFP.AAV) in sterile saline.

For administration of antibodies, the abdomen of the mouse was swabbed with 70% ethanol, and a 30 gauge needle was used to inject prepared antibodies i.p. The aTLR2 antibody was developed using a publicly available TLR2 scFv sequence (27). It was produced as an IgG1 by Sino Biologics (0.2 mg/dose) and administered every 3–4 days.

### Microscopic analysis.

Dissected tissues were fixed in 10% neutral buffered formalin for 48 hours at room temperature, washed twice in 1× PBS and then stored in 70% ethanol solution at 4°C until embedded in paraffin and sectioned into 5 μm slices. Automated IHC was performed on the formalin-fixed paraffin-embedded (FFPE) sections using a Ventana Discovery Ultra automated slide staining system (Roche). Reagents were obtained from Roche and ACDBio (Supplemental Table 1) and used according to manufacturer’s protocol.

Whole-slide scanned images of the stained tissues were acquired using an Aperio CS2 scanner system (Leica). Using Visiopharm Software (Version 2023.09.4.15595x64), images were digitally quantified. Regions of interest (ROIs) encompassing the tissue regions (stained by CK19) were manually drawn. For CD3, Ki67, and GFP, positively stained cells were counted within ROIs, normalized to the ROI areas, and reported as density (cells per mm^2^).

### Flow cytometry.

Peripheral blood was collected via tail vein bleeds or tissues (tumors) were collected from euthanized mice. Tissues were minced into small pieces in DMEM containing collagenase (1 mg/mL, Sigma-Aldrich) and DNase (150 U/mL, Roche). Tissues were incubated at 37°C for 30 minutes, agitated with transfer pipettes, and then incubated for an additional 15 minutes at 37°C. Tissue pieces were strained through 70 μM nylon cell strainers (Corning) to create a single-cell suspension, washed with 10 mL FACs buffer (1× PBS with 2% FBS and 0.2 mM EDTA), and then centrifuged for 8 minutes at 620 × *g*. To remove RBCs, cells were resuspended in 3 mL ACK Lysis Buffer (Life Technologies) for 5 minutes at room temperature, quenched with 5mL FACs buffer, and then centrifuged for an additional 8 minutes at 620 × *g*. Cells were resuspended in 1 mL FACs buffer and strained through 40 μM nylon cell strainers (Corning) to ensure single-cell suspension. Cells were then stained with Trypan Blue and counted using a TC20 automated cell counter (Bio-Rad). Cells were stained with Live/Dead Fixable Aqua Dead Cell stain (Thermo Fisher Scientific), washed with FACs buffer, and then stained with appropriate antibodies (Supplemental Table 2). Finally, cells were fixed in 3% formaldehyde in PBS, washed 3× in FACs Buffer, and data were collected using a FACS Canto II (BD Biosciences). FlowJo (FlowJo, LLC, version 10.9.0) was used to analyze flow cytometry data.

### ELISA.

Peripheral blood was collected via retro-orbital bleeds and left at room temperature until coagulation. The coagulated blood was then centrifuged at 13,000*g* for 20 minutes. The resulting serum was collected for analysis. SAA levels were measured using a mouse SAA1/ SAA2 ELISA kit (Invitrogen, KMA0021), following the manufacturer’s instructions.

### Chimeric antigen receptor T cell production.

Mouse mesothelin CAR plasmid (mmeso.T2A.GFP) was produced as previously described (28) and was gifted from Carl H. June at the University of Pennsylvania. To generate CAR retrovirus, 20 μg of CAR plasmid was packaged in 8 × 10^6^ Plat E cells per flask. T cells were isolated from splenocytes using a mouse T cell isolation kit (STEMCELL Technologies) and activated at a 2:1 ratio (beads/cells) with CD3/CD28 Dynabeads (Gibco, Thermo Fisher Scientific) and recombinant mouse IL-2 (50 U/mL). Fresh media supplemented with mouse IL-2 was replenished daily to maintain activation. Three days after bead stimulation, the cells were transduced with the CAR retrovirus on plates coated with Retronectin recombinant human fibronectin (Takara Bio). CART cells were harvested on day 5 after transduction and immediately used for downstream experiments as mouse CART cells do not survive freeze-thaw cycles.

### AAV production and titration.

AAV vectors were produced and quantified as previously described (29). 293FT cells (Thermo Fisher Scientific) were seeded at 1.5 × 10^7^ cells per flask. On the following day, cells were triple transfected with pHelper, pAAV Rep-Cap, and pAAV expression plasmids in a 1:1:1 molar ratio using polyethyleneimine (Polysciences). Following transfection, the media were replaced twice with DMEM containing 1% FBS. AAV was purified from cell lysates using iodixanol density-gradient ultracentrifugation at 350,000 × *g*. Buffer exchange was performed to 0.01% Pluronic P68 (Sigma-Aldrich) using Amicon Ultra-100 kDa MWCO Centrifugal Filter Units (MilliporeSigma). AAV titers were determined via SYBR Green quantitative polymerase chain reaction (qPCR) using primers targeting the AAV2 ITR (Supplemental Table 3). Vectors were stored at –80°C until use.

### Bulk RNA-seq and analysis.

RNA was extracted from tumors by scraping FFPE tissue sections from slides and isolating RNA using the RNeasy FFPE Kit (Qiagen) according to the manufacturer’s protocol. RNA quality was evaluated using a 2100 Bioanalyzer (Agilent), and libraries were prepared using the Quantseq 3′ mRNA-Seq Library Prep Kit FWD for Illumina (Lexogen). Sequencing was performed on a NextSeq 500 system (Illumina) at the Wistar Institute Genomics Facility. The resulting FASTQ files were uploaded to the Illumina BaseSpace Suite and aligned using the RNA-Seq Alignment application (version 2.0.2), employing STAR for sequence alignment with a maximum mismatch threshold of 14, as recommended by Lexogen. Differential gene expression analysis was conducted with the DESeq2 package. Biological processes enriched in the experimental groups were identified using Gene Ontology analysis with the gprofiler2 package and gene set enrichment analysis (GSEA version 4.2.3).

### Statistics.

Statistical analysis was conducted using Prism software (GraphPad, version 10.4.0). For unpaired group comparisons, unpaired Mann-Whitney *U* tests were applied, while 1-way ANOVA with Tukey’s post hoc test was used for multiple comparisons unless specified otherwise. Kaplan-Meier overall survival curves were compared using Log-rank (Mantel-Cox) tests. *P* values of less than 0.05 were considered significant. Investigators were not blinded to group allocation during experiments or outcome assessment. Unless otherwise stated, data are shown as mean ± SD.

### Study approval.

Animal protocols were reviewed and approved by the IACUC of the University of Pennsylvania, Philadelphia, Pennsylvania, USA (protocol no. 803605).

### Data availability.

Raw human scRNA-seq data and mouse tumor RNA-seq data available in the Gene Expression Omnibus (GEO) under access nos. GSE205013 and GSE213736, respectively, were reanalyzed (30). In addition, data generated from the TCGA Research network (https://www.cancer.gov/tcga) was analyzed and is publicly available through the GDC Data Portal (https://portal.gdc.cancer.gov/). Values for all data points in graphs are reported in the Supporting Data Values file.

## Author contributions

JP, MLS, JCM, and GLB conceived and designed the study. JP, MLS, JCM, HC, KM, KC, JL, and AST performed experiments and collected data. JP, MLS, JCM, HC, KM, KC, JL, AST, DD, and GLB analyzed and interpreted the data. JP, JCM, and GLB drafted the manuscript with input from all authors. GLB supervised the study. All authors revised the manuscript critically for important intellectual content and approved the final version of the manuscript.

## Conflict of interest

JL has received compensation for prior consulting work from Anchor Molecular and Prescient Medicine unrelated to this work. GLB reports active roles as consultant/advisory board member for Hibercell, Pancreatic Cancer Action Network, EMD Serono, and Alligator Biosciences; reports receiving active research funding from Alligator Biosciences, Incyte Corporation, and Bristol-Myers Squibb; and is an inventor of intellectual property related to CAR T cells that is managed by the University of Pennsylvania.

## Funding support

This work is the result of NIH funding, in whole or in part, and is subject to the NIH Public Access Policy. Through acceptance of this federal funding, the NIH has been given a right to make the work publicly available in PubMed Central.

- NIH grants R01-CA197916 (GLB), R01-CA245323 (GLB), F31CA271692 (JP), and T32CA251063 (JCM)
- Department of Defense grants W81XWH2110622 (MLS) and W81XWH2110621 (GLB)
- 2020 AACR-The Mark Foundation for Cancer Research “Science of the Patient” (SOP) Grant (20-60-51-BEAT)
- Research grant from the Lustgarten Foundation Inc.

## Supplementary Material

Supplemental data

Unedited blot and gel images

Supporting data values

## Version 1 04/03/2026

In-Press Preview

## Version 2 05/22/2026

Electronic publication
