# Supplementary material for: TLR2 signaling regulates T cell exclusion in pancreatic ductal adenocarcinoma
Source: JCI Insight. 2026 Mar 31;11(10):e195329. doi: 10.1172/jci.insight.195329 (PMC13232717; doi:10.1172/jci.insight.195329)
Supplement: Supplemental data [file jciinsight-11-195329-s017.pdf]

TLR2 signaling regulates T cell exclusion in pancreatic ductal adenocarcinoma

By Jacqueline Plesset, Meredith L. Stone, John C. McVey, Heather Coho, Kelly Markowitz, Kay Coho, Jesse Lee, Anna S. Thickens, Devora Delman, Gregory L. Beatty

SUPPLEMENTAL MATERIALS

Supplemental Figure 1

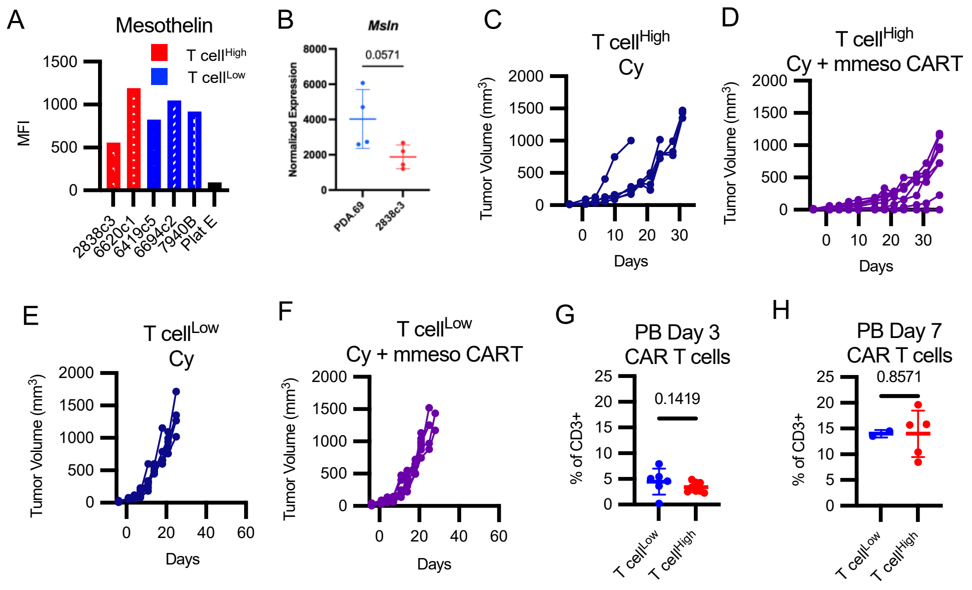

Supplemental Figure 1. Mesothelin expression and CART cell dynamics in T cell^{High} and T cell^{Low} PDAC models. A) Mesothelin MFI expression in T cell^{High} and T cell^{Low} cell lines. Plat E is a engineered 293T cell which does not express mesothelin. **B)** *Msln* expression measured by qRT-PCR in tumors derived *in vivo* from PDA.69 (T cell^{Low}) compared to 2838c3 (T cell^{High}). Mann Whitney test performed. **C**- **F**) Mice were treated as in Figure 1B. Individual tumor curves . **G)** Day 3 and **H**) Day 7 of CAR T cell expansion in peripheral blood from mice who received either T cell^{High} or T cell^{Low} tumors (Mann Whitney test performed).

Supplemental Figure 2

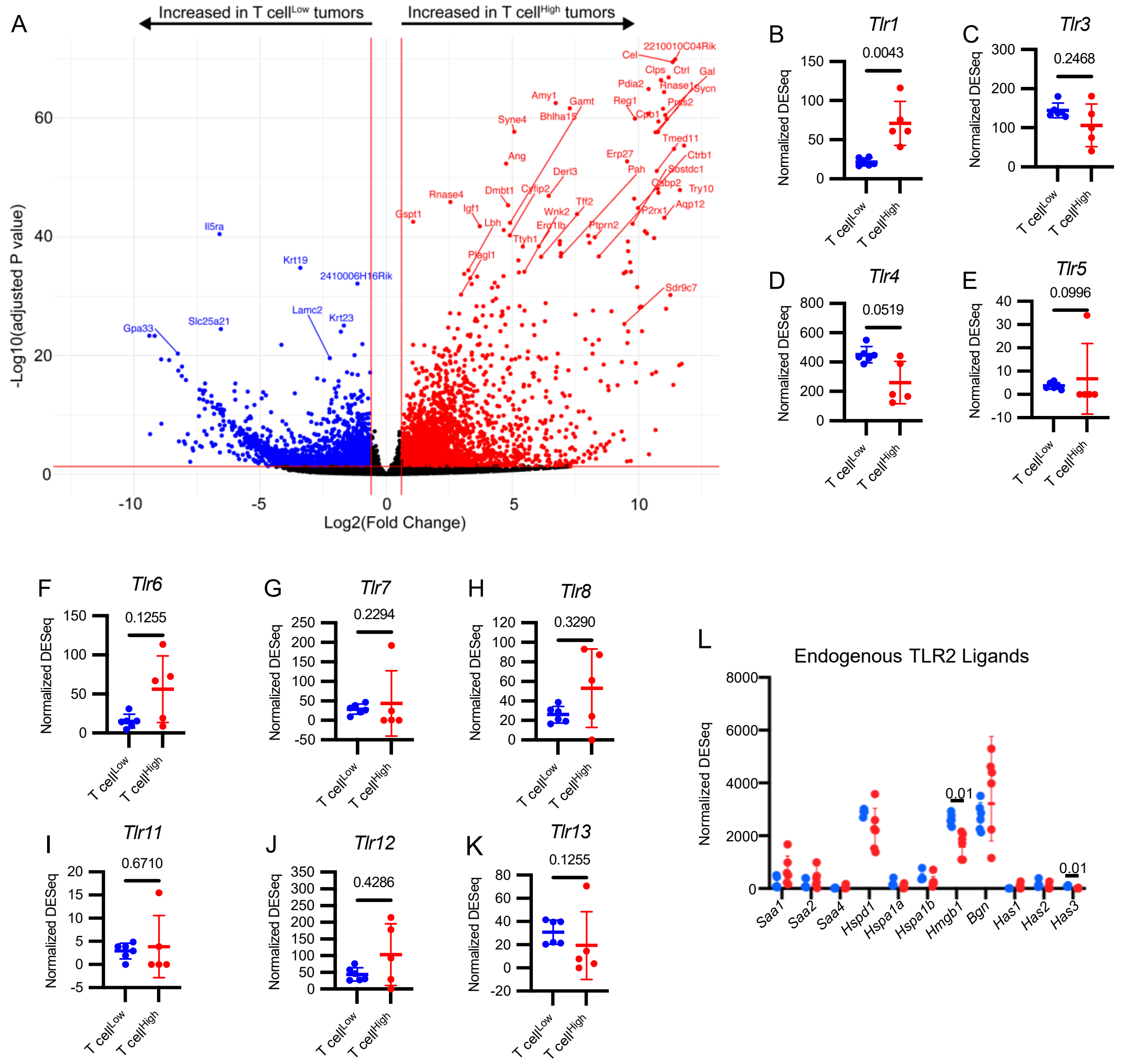

Supplemental Figure 2. Bulk RNA sequencing in T cell^{High} and T cell^{Low} mouse PDAC. A) Volcano plot showing upregulated genes in T cell^{High} and T cell^{Low} tumors. **B)** Normalized *Tlr1*, **C)** *Tlr3*, **D)** *Tlr4*, **E)** *Tlr5*, **F)** *Tlr6*, **G)** *Tlr7*, **H)** *Tlr8*, **I)** *Tlr11*, **J)** *Tlr12*, **K)** *Tlr13* DESeq expression from bulk RNA-seq data from Figure 2A-C (Mann Whitney tests performed). **L)** Normalized endogenous TLR2 ligands DESeq expression from bulk RNA-seq data from Figure 2A-C (Mann Whitney test with correction for multiple comparisons performed).

Supplemental Figure 3

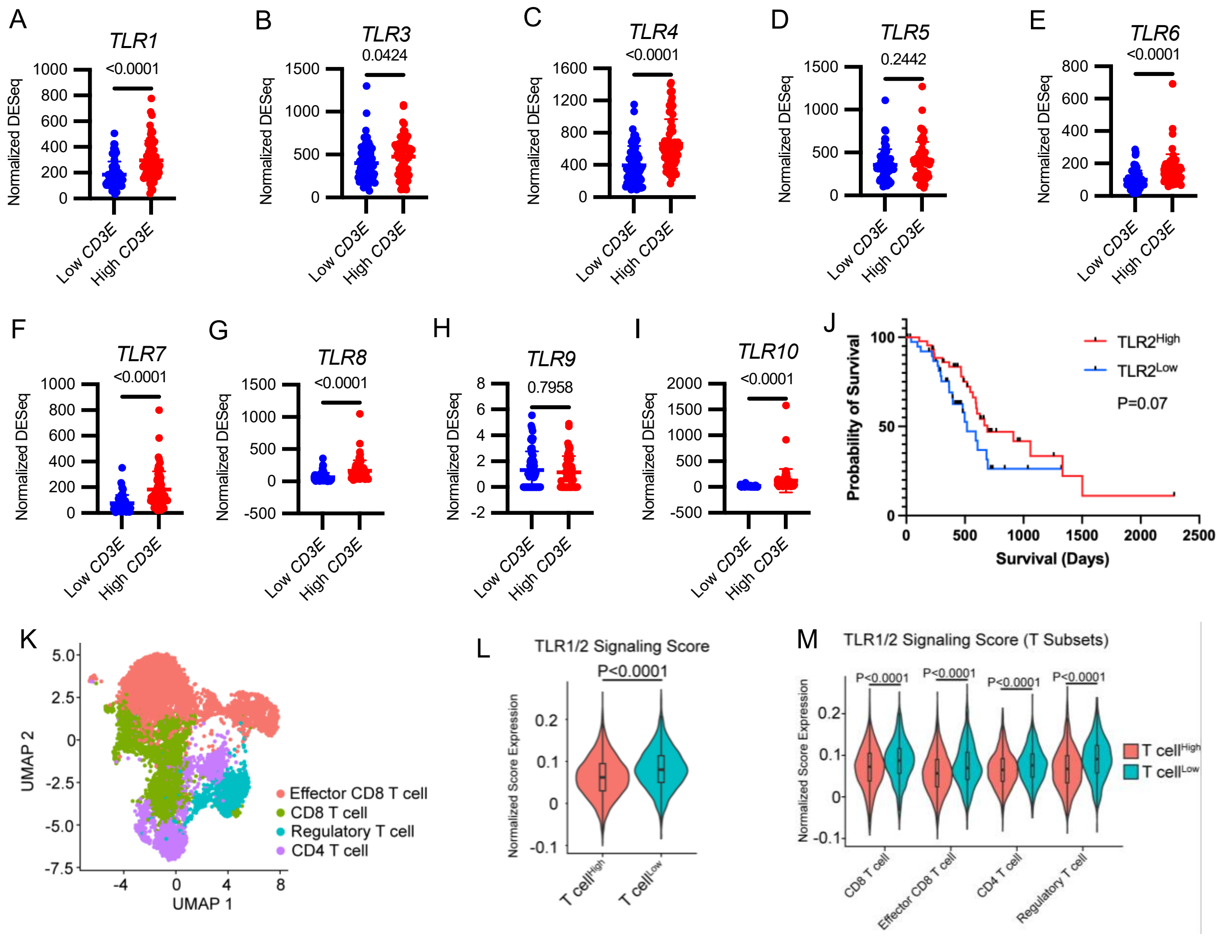

Supplemental Figure 3. TLR molecule expression and signaling in human PDAC. A) Normalized *TLR1*, **B)** *TLR3*, **C)** *TLR4*, **D)** *TLR5*, **E)** *TLR6*, **F)** *TLR7*, **G)** *TLR8*, **H)** *TLR9*, **I)** *TLR10* RNA expression of high (quartiles 3,4) versus low (quartiles 1,2) *CD3E* gene expression from TCGA data in Figure 2H-J. Mann Whitney tests performed. **J)** Kaplan-Meier curve showing overall survival of high CD3E tumor expressing patients subdivided by $TLR2^{High}$ and $TLR2^{Low}$ expression. Log-Rank test performed. **K)** UMAP clustering showing T cell population from Figure 2L. **L)** TLR1/2 signaling score in T cells between T $cell^{High}$ and T $cell^{Low}$ tumors. TLR1/2 signaling score derived from 115 genes in the “Reactome Toll Like Receptor TLR1 TLR2 Cascade” gene set. Mann Whitney test performed. **M)** TLR1/2 signaling score between T cell subsets identified in J. Mann Whitney test performed.

Supplemental Figure 4

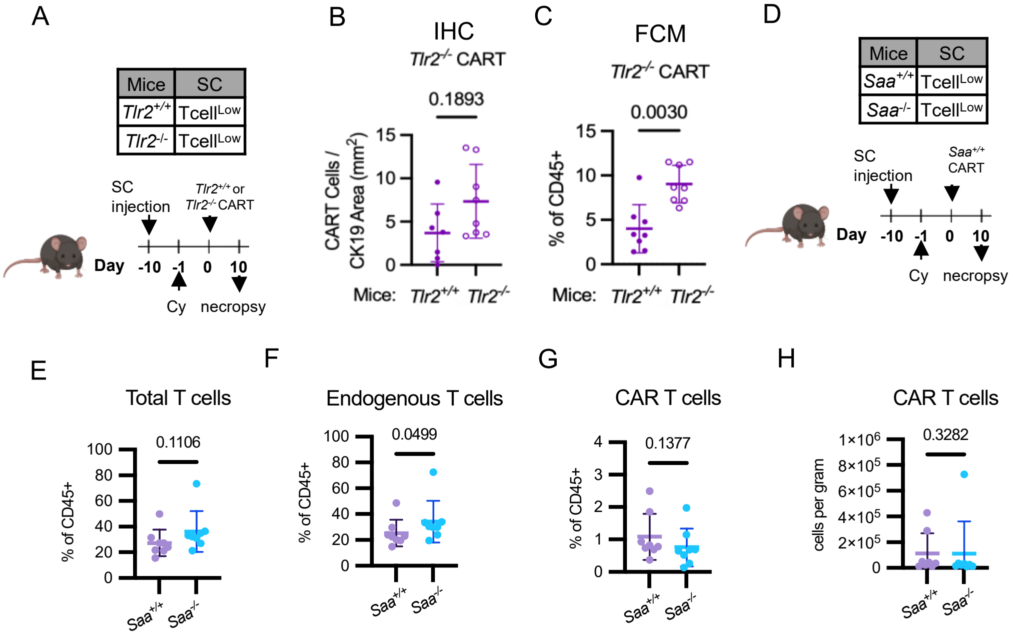

Supplemental Figure 4. Impact of SAA and TLR2 deficiency on endogenous T cell and meso-CAR T cell infiltration in T cell^{Low} PDAC model. A) Study design of G and H. T cell^{Low} cells (1e6) were implanted subcutaneously into *Saa*$^{+/+}$ and *Saa*$^{-/-}$ mice on day -10. Each group consisted of 8 mice. On day -1, mice received cyclophosphamide (120mg/kg dose, i.p.) and received meso *Tlr2*$^{+/+}$ or *Tlr2*$^{-/-}$ CAR-T cells (5-8e6 cells/mouse, i.v.) on days 0 with necropsy 10 days later. **B)** Immunohistochemistry showing Tlr2$^{-/-}$ CAR-T cell infiltration into PDAC tumors of *Tlr2*$^{+/+}$ and *Tlr2*$^{-/-}$ mice. **C)** Flow cytometry showing Tlr2$^{-/-}$ CAR-T cell infiltration into PDAC tumors of *Tlr2*$^{+/+}$ and *Tlr2*$^{-/-}$ mice. **D)** Study design of B-E. T cell^{Low} cells (1e6) were implanted subcutaneously into *Saa*$^{+/+}$ and *Saa*$^{-/-}$ mice on day -10. Each group consisted of 8 mice. On day -1, mice received cyclophosphamide (120mg/kg dose, i.p.) and received meso *Saa*$^{+/+}$ CAR-T cells (5-8e6 cells/mouse, i.v.) on days 0 with necropsy 10 days later. **E)** Analysis of Total T cell, **F)** Endogenous T cell, and **G)** CAR T cell percentages in the tumors via flow cytometry (Mann Whitney tests performed). **H)** Analysis of CAR T cells per gram in the tumors via flow cytometry (Mann Whitney tests performed).

Supplemental Figure 5

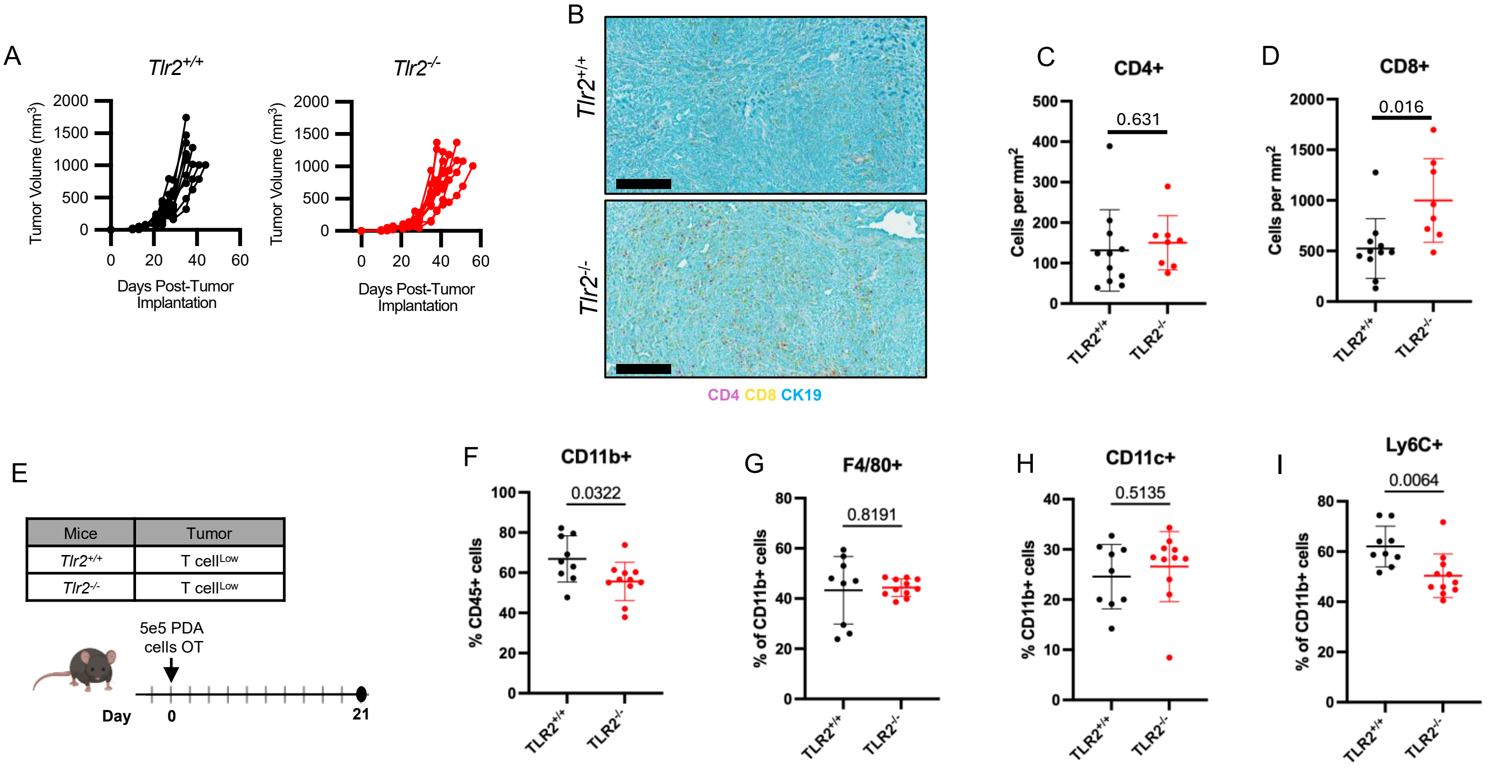

Supplemental Figure 5. T cell and myeloid cell profiling of T cell^{Low} tumors in Tlr2$^{+/+}$ vs Tlr2$^{-/-}$ mice. A) Mice were treated as in Figure 4A. Individual tumor curves are shown. **B)** Representative images of tumors stained for CD4 (pink), CD8 (yellow) and CK19 (blue). Scale bar, 300µm. **C)** Analysis of T cell (CD4 stained) per tumor area (CK19 stained) from A (Welch's test performed). **D)** Analysis of T cell (CD8 stained) per tumor area (CK19 stained) from A (Welch's test performed). **E)** Study schematic for F-G. T cell^{Low} (PDA.69) cells (5e5) were implanted orthotopically into *Tlr2*$^{+/+}$ or *Tlr2*$^{-/-}$ mice (n=9-11 mice/group) on Day 0. Data shown are representative of n=1 biological replicates. **F)** Analysis of CD11b+ myeloid cells as a percentage of CD45 cells in the tumors via flow cytometry (Mann Whittney test performed). **G)** Analysis of F4/80+ macrophages as a percentage of CD11b+ cells in the tumors via flow cytometry (Mann Whittney test performed). **H)** Analysis of CD11c+ dendritic cells as a percentage of CD11b+ cells in the tumors via flow cytometry (Mann Whittney test performed). **I)** Analysis of LY6c+ monocytes as a percentage of CD11b+ cells in the tumors via flow cytometry (Mann Whittney test performed).

Supplemental Figure 6

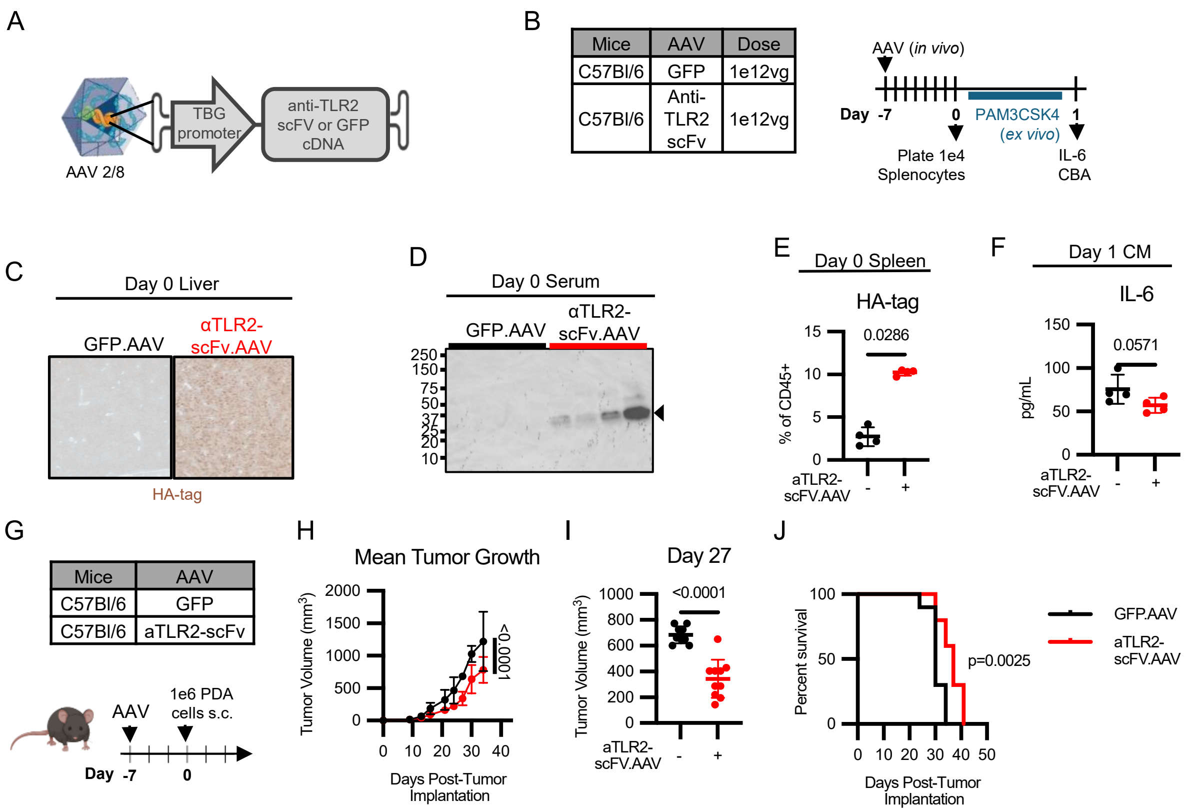

Supplemental Figure 6. Characterization and efficacy of TLR2-blocking scFv in PDAC model. A) Design of an AAV to express a TLR2 blocking scFv with an HA tag (αTLR2-scFv) in hepatocytes. **B)** Study schematic. Mice were treated with 1e12 viral genomes (vg) of the AAVs indicated. 7 days later, splenocytes were harvested and treated *ex vivo* with TLR2 agonist PAM3CSK4 (1000ng/mL) **C)** Immunohistochemistry showing expression of αTLR2-scFv in hepatocytes of mice treated as in (B). Livers were stained for HA. **D)** Western blot detecting αTLR2-scFv in serum of mice treated as in (B). Membranes were blotted for HA. Arrow indicates anti-TLR2-scFv at 27 kDa. **E)** Spleens were analyzed by flow cytometry for binding of αTLR2-scFv to splenocytes. scFv binding is accessed by detection of HA. **F)** IL-6 in media of splenocytes harvested from mice as in (B) and treated with PAM3CSK4 overnight. **G)** Study schematic. Mice were treated with AAVs (GFP or aTLR2-scFv). 7 days later, T cell^{Low} tumor cells were subcutaneously implanted. Mice were monitored for overall survival. **H**) Mean tumor growth curves (two-way ANOVA test performed) **I)** Tumor volume Day 27 (Mann Whitney test performed). **J)** Overall survival (Mantel Cox test performed).

Supplemental Figure 7

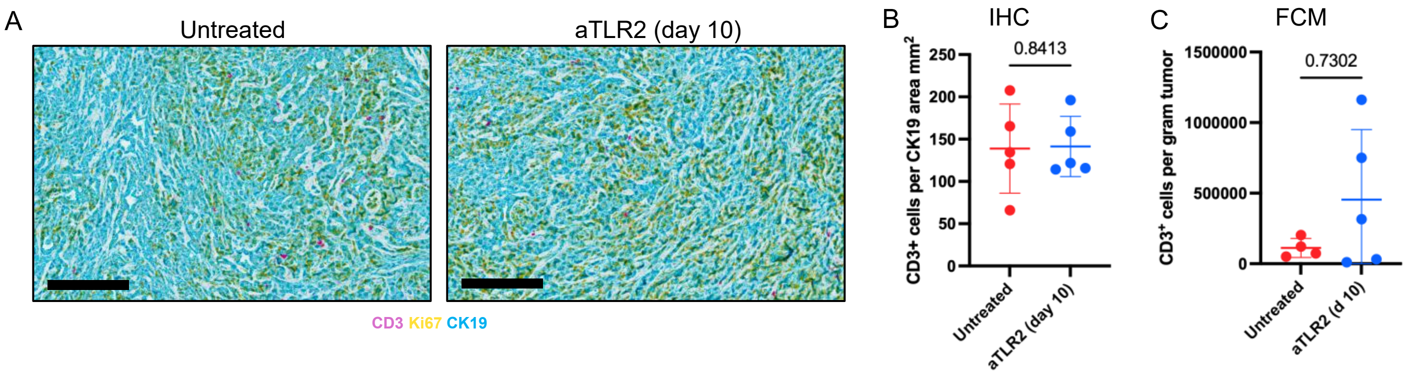

Supplemental Figure 7. Characterization of T cell tumor infiltration in therapeutic anti-TLR2 antibody treatment. A) Representative IHC images of untreated and anti-TLR2 treated mice. Anti-TLR2 treatment started on day 10 and was dosed at 0.2mg every 3-4 days. Tumors were collected on day 15. **B)** Quantification of A. Mann-Whitney test performed. **C)** Flow cytometry of tumors staining for $CD3^+$ cells from untreated and anti-TLR2 treated mice (day 10). Mann-Whitney test performed.

Supplemental Table 1

Application	Target Antigen/ Cell		Target Species	Host Species	Clone	Conjugation	Dilution	Vendor	Catalog #
Automated IHC	Primary Antibody	CD3	Mouse	Rabbit	Polyclonal	Unconj.	1:200	Abcam	ab5690
		Ki67	Mouse	Rabbit	D3B5	Unconj.	1:200	Cell Signaling	12202
		CK19	Mouse	Rabbit	EPNCIR127B	Unconj.	1:800	Abcam	ab133496
		GFP	Mouse	Goat	Polyclonal	Unconj.	1:300	Abcam	ab6673
		HA	Mouse	Chicken	Polyclonal	Unconj.	1:800	Abcam	ab1190
	Secondary Antibody	HQ	N/A	N/A	N/A	HRP	No dilution	Roche	760-4820
		IgG	Rabbit	Goat	Polyclonal	NP	No dilution	Roche	760-4817
		IgG	Rabbit	Goat	Polyclonal	HQ	No dilution	Roche	760-4815
		NP	N/A	Mouse	Monoclonal	AP	No dilution	Roche	760-4827
		IgG	Goat	N/A	N/A	HRP	No dilution	Roche	760-4647
		IgG	Chicken	Rabbit	Polyclonal	HRP	1:3000	Jackson ImmunoResearch	303-035- 003

Chromogen	Application(s) used	Vendor	Catalog #
Purple	Automated IHC	Roche	760-229
Teal	Automated IHC	Roche	760-247
Yellow	Automated IHC	Roche	760-239
DAB (Brown)	Automated IHC	Roche	760-159

Supplemental Table 1: Antibodies and reagents for immunohistochemistry.
IHC, immunohistochemistry

Supplemental Table 2

Application	Target Antigen/ Cell	Target Species	Host Species	Clone	Conjugation	Dilution	Vendor	Catalog #
FCM	CD11b	Mouse	Rat	M1/70	PerCp	1:100	BD Biosciences	561114
	CD11c	Mouse	Hamster	HL3	PE-Cy7	1:100	BD Biosciences	558079
	CD11c	Mouse	Hamster	N418	APC	1:100	BioLegend	117310
	CD11c	Mouse	Hamster	N418	FITC	1:100	BioLegend	117306
	CD19	Mouse	Rat	6D5	PB	1:100	BioLegend	115523
	CD3	Mouse	Rat	17A2	PE-Cy7	1:100	BioLegend	100220
	CD3	Mouse	Rat	17A2	PB	1:100	BioLegend	100214
	CD45	Mouse	Rat	30-F11	PerCp	1:100	BD Biosciences	550994
	CD45	Mouse	Rat	30-F11	PE-Cy7	1:100	BD Biosciences	552848
	CD45	Mouse	Rat	30-F11	APC-Cy7	1:100	BD Biosciences	561037
	CD45.1	Mouse	Mouse	A20	APC-Cy7	1:100	BioLegend	110716
	CD45.2	Mouse	Mouse	104	PE-Cy7	1:100	BioLegend	109830
	F4/80	Mouse	Rat	BM8	PB	1:100	BioLegend	123124
	F4/80	Mouse	Rat	BM8	APC	1:100	BioLegend	123116
	F4/80	Mouse	Rat	BM8	APC-Cy7	1:100	BioLegend	123118
	Goat anti-Rat	Rat	Goat	Poly4054	PE-Cy7	1:100	BioLegend	405413
	HA	N/A	Mouse	16B12	PE-Cy7	1:100	BioLegend	901528
	Ly6C	Mouse	Rat	HK1.4	PB	1:100	BioLegend	128014
	Ly6C	Mouse	Rat	HK1.5	FITC	1:100	BioLegend	128006
	Ly6G	Mouse	Rat	1A8	PB	1:100	BioLegend	127612
	Mesothelin	Mouse	Rat	B35	Unconjugated	1:200	Isbio	LS-C179484-100
	TLR2	Mouse	Rat	CB225	PE	1:100	BioLegend	148604

Supplementary Table 2: Antibodies for flow cytometry.
FCM, flow cytometry

Supplemental Table 3

Gene	Forward Sequence	Reverse Sequence
ltr	GGAACCCCTAGTGATGGAGTT	CGGCCTCAGTGAGCGA

Supplementary Table 3: Primer sequences.

Supplemental Table 4

Supplemental Table 4: Top 100 differentially expressed genes between T cell^{Low} vs T cell^{High} tumors.

Gene ID	Log2(Fold Change)	Log2(Fold Change) SE	P value	Adjusted P value
Hamp2	10.02095329	0.422064518	1.31E-124	2.13E-120
Cela2a	11.78088221	0.574808499	2.37E-93	1.93E-89
Cela1	10.98340098	0.543259789	6.86E-91	3.72E-87
Cckar	10.92199865	0.578754836	1.95E-79	7.95E-76
Tmed6	9.280159594	0.506629646	6.01E-75	1.96E-71
Pla2g1b	10.70418347	0.585181145	9.57E-75	2.60E-71
Chst2	6.209435988	0.340818529	3.64E-74	8.45E-71
2210010C04Rik	11.45159128	0.629720499	6.76E-74	1.38E-70
Cel	11.3556429	0.626607695	2.12E-73	3.83E-70
Ctrl	11.19432734	0.629322607	8.77E-71	1.43E-67
Clps	10.88934166	0.614334038	2.67E-70	3.95E-67
Pdia2	10.39605771	0.593266509	9.49E-69	1.29E-65
Rnase1	11.01596442	0.631258013	3.39E-68	4.25E-65
Amy1	6.718569909	0.390620217	2.67E-66	3.10E-63
Bhlha15	7.274057322	0.42596115	2.21E-65	2.40E-62
Cpb1	10.96876012	0.642755894	2.69E-65	2.74E-62
Cuzd1	10.382839	0.612639126	2.00E-64	1.91E-61
Gal	11.05718312	0.653728186	3.55E-64	3.21E-61
Reg1	9.861482965	0.585964648	1.48E-63	1.27E-60
Zg16	11.12281637	0.661386773	1.82E-63	1.48E-60
Cpa1	10.78570951	0.643584756	4.88E-63	3.78E-60
Syne4	5.071715152	0.30712988	2.95E-61	2.18E-58
Sycn	10.76897142	0.652356836	3.22E-61	2.28E-58
Prss2	10.67254483	0.646807686	3.65E-61	2.47E-58
Try4	11.80423965	0.729445636	6.70E-59	4.36E-56
Pnlip	11.40934665	0.708745891	2.64E-58	1.65E-55
1810018F18Rik	12.44226346	0.781806062	5.01E-57	3.02E-54
Erp27	9.548608753	0.604634962	3.51E-56	2.04E-53
Ang	4.75131454	0.30191297	8.39E-56	4.71E-53
Tmed11	10.72420241	0.689581909	1.55E-54	8.40E-52
Cabp2	10.76565493	0.712650495	1.47E-51	7.70E-49
Try10	11.64154875	0.772684462	2.70E-51	1.37E-48
Cpa2	10.78649867	0.71946553	8.24E-51	4.06E-48
Derl3	6.441117489	0.431961884	2.78E-50	1.33E-47
Slc38a5	9.826867207	0.662277343	8.32E-50	3.87E-47
Rnase4	2.540181349	0.172219139	3.09E-49	1.40E-46
Dmbt1	4.828967161	0.329394293	1.16E-48	5.10E-46
Ctrb1	9.982584948	0.684387484	3.44E-48	1.47E-45
1810010D01Rik	7.560580508	0.524144274	3.62E-47	1.51E-44
Aqp12	11.02465064	0.769884833	1.64E-46	6.69E-44
Gspt1	1.066570157	0.075037426	7.52E-46	2.98E-43
Gamt	4.909675923	0.346177861	1.18E-45	4.55E-43
Sostdc1	9.766803203	0.689851165	1.67E-45	6.32E-43
Igf1	3.707953555	0.263236943	4.63E-45	1.71E-42
ambiguous	4.654445208	0.332923422	2.05E-44	7.41E-42
Cela3b	10.25173583	0.735059491	3.29E-44	1.16E-41
Spink1	10.33290522	0.744301246	8.07E-44	2.79E-41
Il5ra	-6.610190489	0.47671447	1.02E-43	3.44E-41
Cyfip2	4.899417975	0.354304292	1.72E-43	5.72E-41
Dusp26	8.004555857	0.579278746	1.98E-43	6.45E-41
Ptprn2	8.265879555	0.600253568	3.83E-43	1.22E-40
Cela3a	10.61873677	0.77254428	5.45E-43	1.70E-40
Cldn10	6.886072606	0.50401335	1.70E-42	5.22E-40
Pnliprp1	9.582680966	0.702252179	2.14E-42	6.46E-40
Hsd17b13	8.054333343	0.591797934	3.50E-42	1.03E-39
Egf	6.877982612	0.507099016	6.60E-42	1.92E-39
Ero1lb	6.049735999	0.448000311	1.48E-41	4.24E-39
Ttyh1	5.413192941	0.401014581	1.59E-41	4.46E-39
Klk1b5	9.587301001	0.717423392	9.88E-41	2.72E-38
Fam174b	6.92876811	0.520688709	2.11E-40	5.72E-38
Pah	6.915388572	0.523565532	7.86E-40	2.10E-37
P2rx1	8.427221588	0.638100595	8.02E-40	2.11E-37
Tff2	6.138213786	0.465251646	9.59E-40	2.48E-37
Prss8	5.236457417	0.399995371	3.69E-39	9.39E-37
Klk1	9.664382013	0.741679554	8.22E-39	2.06E-36
Krt19	-3.408891696	0.26494253	6.95E-38	1.71E-35
Lbh	3.252310488	0.254307271	1.89E-37	4.59E-35
Wnk2	5.47196898	0.429379292	3.37E-37	7.95E-35
Amy2b	9.702994433	0.76135428	3.35E-37	7.95E-35
Klk1b11	9.502666461	0.74589067	3.54E-37	8.24E-35
Casp9	5.256417051	0.413157497	4.43E-37	1.02E-34
Gp2	9.41380807	0.741501688	6.25E-37	1.41E-34
Cachd1	3.091499835	0.243942017	8.34E-37	1.86E-34
Ggh	4.649057312	0.368870424	2.02E-36	4.44E-34
Hnmt	3.604358789	0.286256839	2.36E-36	5.11E-34
Mgll	3.32838687	0.265436522	4.55E-36	9.74E-34
Nupr1	4.299310321	0.346762806	2.67E-35	5.63E-33
2410006H16Rik	-1.143641083	0.092460621	3.85E-35	8.03E-33
Tbc1d30	3.38265381	0.273772897	4.54E-35	9.35E-33
Slc39a5	4.422754308	0.360478627	1.33E-34	2.70E-32
Klk1b3	9.949883329	0.81173814	1.53E-34	3.08E-32
Edem2	4.189907618	0.342375952	1.95E-34	3.88E-32
Plagl1	2.962957841	0.246551322	2.87E-33	5.63E-31
Ctrc	11.26329808	0.938366504	3.42E-33	6.63E-31
Cbs	4.349952272	0.365988781	1.41E-32	2.70E-30
Sel1l	4.219819241	0.357253593	3.39E-32	6.42E-30
Klf15	6.316787626	0.538223789	8.30E-32	1.55E-29
Slc7a8	5.291087842	0.454603097	2.61E-31	4.83E-29
Anpep	3.843262484	0.330306137	2.72E-31	4.98E-29
Mknk1	3.57508864	0.307315302	2.79E-31	5.05E-29
Try5	10.09596579	0.86904932	3.37E-31	6.02E-29
Prom2	10.11917382	0.871439067	3.58E-31	6.33E-29
Pnliprp2	10.0392399	0.866557234	4.90E-31	8.57E-29
Fam46c	4.836827863	0.41792858	5.63E-31	9.74E-29
Klk1b8	11.09353054	0.960958808	7.89E-31	1.35E-28
Pnmal2	5.483950506	0.47757171	1.61E-30	2.72E-28
Lrrc7	8.197481507	0.714059117	1.66E-30	2.79E-28
Gabra4	8.927151588	0.779138345	2.15E-30	3.57E-28
Ptger3	4.599637381	0.401659053	2.31E-30	3.79E-28
Angpt1	2.531806575	0.222223697	4.53E-30	7.37E-28

Supplemental Table 5

Characteristics of the cohort	
n=179	
**Demographic information**	
Age	65 [57-73]
Sex (Male)	98 (54.7)
Race	
African American	7 (3.9)
Asian	10 (5.6)
Hispanic	5 (2.8)
White	157 (84.9)
Not reported	5 (2.8)
**Tumor characteristics**	
T stage	
T1	6 (3.4)
T2	23(12.8)
T3	144 (80.4)
T4	4 (2.2)
Not reported	2 (1.2)
N stage	
N0	48 (26.8)
N1	127 (70.9)
Not reported	4 (2.3)
M stage	
M0	81 (45.3)
M1	5 (2.8)
Not reported	93 (52.0)
Location	3 (60)
Head of pancreas	132 (73.7
Body of pancreas	15 (8.4)
Tail of pancreas	13 (7.3)
Overlapping lesion	2 (1.1)
Not specified	17 (9.5)
Note: Continuous data is presented as mean ± standard deviation while count data is shown as number (%) or median [IQR].	

Supplemental Table 5: Patient characteristics of dataset used for analysis of patients with pancreas cancer.
