# Supplementary figures and images for: TLR2 signaling regulates T cell exclusion in pancreatic ductal adenocarcinoma
Source: JCI Insight. 2026 Mar 31;11(10):e195329. doi: 10.1172/jci.insight.195329 (PMC13232717; doi:10.1172/jci.insight.195329)

Full unedited gel for Supplemental Figure 6D

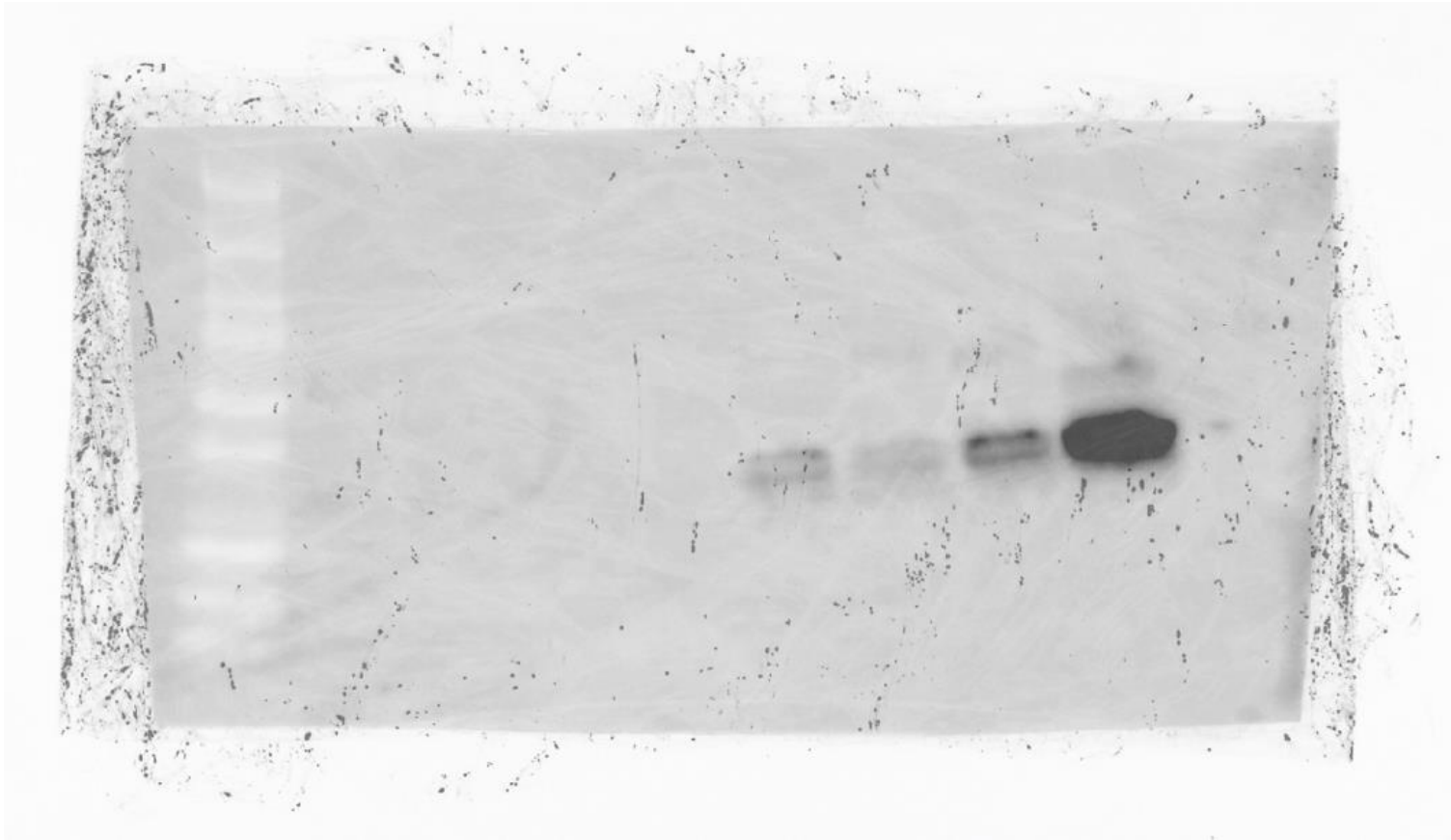

Supplement: Unedited blot and gel images [file jciinsight-11-195329-s018.pdf]
